# Impacts of ultrasonication time and surfactants on stability and optical properties of CuO, Fe_3_O_4,_ and CNTs/water nanofluids for spectrum selective applications

**DOI:** 10.1016/j.ultsonch.2022.106079

**Published:** 2022-06-22

**Authors:** Muhammad Usman Sajid, Yusuf Bicer

**Affiliations:** Division of Sustainable Development (DSD), College of Science and Engineering (CSE), Hamad Bin Khalifa University (HBKU), Qatar Foundation (QF), Education City, Doha, Qatar

**Keywords:** Absorbance, Irradiance, Spectrum, Surfactants, Transmittance

## Abstract

The prime objective of the present experimental work is to evaluate the impact of ultrasonication time and surfactants on the optical characteristics (transmittance and absorbance) and stability of CuO/water, CNTs/water, and Fe_3_O_4_/water nanofluids to be used in spectrum selective applications. Two-step method with various ultrasonication times (30 min, 60 min, and 90 min) was employed to prepare nanofluids (having volume fractions of 0.004 % and 0.0004 %). Furthermore, various surfactants (anionic, cationic, and polymer) were added to the base fluid. The study results revealed that surfactants have a significant effect on the stability of nanofluids over ultrasonication time. The nanofluids prepared using sodium dodecylbenzene sulfonate (SDBS) have the highest zeta potential values than other surfactants used in the experimentation. The increase in transmittance of nanofluid was more prominent for lower concentration (0.0004 %) after one week of preparation. The concentration of nanoparticles, ultrasonication time, temperature, and surfactants influenced the optical characteristics of nanofluids. The most stabled CNTs nanofluid with 0.004 % concentration and 90 min of ultrasonication obtained an average of 67.6 % and 74.6 % higher absorbance than stabled CuO and Fe_3_O_4_ nanofluids, respectively. The irradiance transmitted through nanofluid was strongly dependent on the concentration and type of nanoparticles.

## Highlights

• Preparation of CuO/water, CNTs/water, and Fe_3_O_4_/water nanofluids.
• Effects of ultrasonication time and surfactants on nanofluids stability and optical properties.
• The stability was dependent on the surfactants than ultrasonication time.
• The transmittance of nanofluids increased with the rise in temperature and passage of time.
• Potential applications on the base of spectrum splitting characteristics of nanofluids.

## Nomenclature

ALS: Ammonium lauryl sulfate
BAC: Benzalkonium chloride
CNTs: Carbon nanotubes
CTAC: Cetrimonium chloride
CTAB: Cetyltrimethylammonium bromide
f-MWCNTs: Functionalized multi-walled carbon nanotubes
GA: Gum Arabic
MWCNTs: Multi-walled carbon nanotubes
PVP: Polyvinylpyrrolidone
PLS: Potassium lauryl sulfate
SDS: Sodium dodecyl sulfate
SDBS: Sodium dodecylbenzene sulfonate
TMAH: Tetramethylammonium hydroxide

**Greek Symbols**
∅: Volume fraction
ρ: Density

## 1 Introduction

The nanofluid contains nano-sized particles (usually 1 – 100 nm) disseminated in conventional fluids like water, ethylene glycol, propylene glycol, thermal oil, etc. This insertion of nanoparticles (which may be made of metals, non-metals, oxides, or other compounds) in the traditional fluids affects their thermo-physical properties [1] and alters the optical characteristics of base fluids. The enhanced heat transfer performance of nanofluids made them a prospective candidate for miscellaneous applications, including heat sinks [2], radiators [3], heat exchangers [4], greenhouse cooling [5], and solar collectors [6], etc. The stability of nanoparticles in the base fluid plays a critical role in improving nanofluids' effective thermal conductivity and optical properties. Due to the high specific energy of nanoparticles, the agglomeration and sedimentation of nanoparticles can take place after a particular period. This clustering and settlement of nanoparticles have an adverse impact on the heat transfer performance of nanofluids. The ultrasonication process provides a viable solution by breaking up the aggregated nanoparticles and uniformly dispersing them in the base fluid [7].

The ultrasonication process can be divided into two categories. One is direct sonication, in which the ultrasonic probe is immersed in the colloidal mixture, and the other is indirect sonication, where the ultrasonic waves are transmitted to submerged nanofluid samples through the liquid. It is important to note that there is no specified time period for sonication of nanofluids, and different studies used various sonication times for similar nanofluids. Table 1 summarizes the type of nanofluids and sonication times used during the preparation of similar nanofluids in the literature.

**Table 1:** Sonication time used for similar nanofluids in the literature.

Study	Nanofluid	Concentration range	Sonication time (Hours)	Amplitude
[8]	TiO_2_/water	0.1 – 0.3 vol%	4	–
[2]	TiO_2_/water	0.006 – 0.012 vol%	3	–
[9]	TiO_2_/water	0.5 vol%	2.5	50 %
[10]	TiO_2_/water	0.05 – 0.15 vol%	1	–
[11]	TiO_2_/water	1.25 vol%	0.5	–
[12]	Al_2_O_3_/water	0 – 1 vol%	2 – 3	–
[13]	Al_2_O_3_/water	0.1 and 0.3 wt%	3	–
[14]	Al_2_O_3_/water	0.01 – 0.08 vol%	1	–
[15]	Al_2_O_3_/water	0.1, 0.5 and 1.0 wt%	0.33	30 %
[16]	MgO/water	5 vol%	8	–
[17]	MgO/water	0.07 – 1.25 vol%	6	–
[18]	MgO/water	0.006–0.01 vol%	2	–
[19]	CuO/water	0.015 vol%	0.5	–
[20]	CuO/Transformer oil	0.01−0.04 vol%	2	–

Limited studies are available in the literature that evaluated the impact of sonication time on the stability of nanofluids. Mahbubul et al. [7] studied the effects of ultrasonication time on the stability of Al_2_O_3_ nanoparticles in the water. The ultrasonication time varied between 0 and 5h. The highest value of zeta potential was observed for 3h of ultrasonication. Studies [[21], [22]] also showed similar results for Al_2_O_3_ nanofluids. The thermal conductivity and stability of alumina nanofluid decreased for ultrasonication time of more than 5h [23]. Safiei et al. [24] suspended Al_2_O_3_ nanoparticles in water and ethylene glycol mixture having various ratios (80:20, 70:30, and 60:40). The results revealed that nanofluid with a mixture ratio of (60:40) displayed the highest stability when the sonication time was 3h. The most stable TiO_2_/water nanofluid was yielded at a sonication duration of 2.5h [9]. The TiO_2_ nanoparticles started to aggregate in the water when the ultrasonication duration exceeded 2.5 h.

Sonawane and Juwar [25] found that optimal sonication time for Fe_3_O_4_/ethylene glycol nanofluid was 3.6h. The thermal conductivity was maximum with minimum viscosity at the optimal sonication time. Zheng et al. [26] varied the sonication time of Fe_3_O_4_/liquid paraffin nanofluid between 2 and 4h. The results showed that 3h of sonication produced the best stability of nanoparticles in the base fluid. The optimal sonication was not function of temperature and volume fraction of nanoparticles. Chen et al. [27] explored the influence of sonication duration on the stability and thermal conductivity of Al_2_O_3_/paraffin nanofluid stabilized by oleic acid. The thermal conductivity of nanofluid elevated up to sonication time of 3.25h, beyond which a decrease in thermal conductivity was observed due to breakage of bondage between nanoparticles and surfactant. Similar results were obtained in a study [28] where MWCNTs/water nanofluid thermal conductivity and stability enhanced until 1h of ultrasonication, and further ultrasonication resulted in a deterioration of stability and thermal conductivity. Jozwiak et al. [29] noticed a decrease in thermal conductivity of MWNCTs ionanofluids with the increase in ultrasonication time due to breakage of particle clusters. Said et al. [30] found the optimal ultrasonication time for f-MWCNTs nanofluids. The study results showed that the samples prepared using 80 min of ultrasonication attained the highest stability. The ultrasonication time has a dominating impact on the stability of few-layer graphene nanofluid than the ultrasonication power [31]. The thermal conductivity of new transformer oil containing CuO-nanostrips was higher than the old transformer oil for the same sonication time of 2h [32].

Further, the addition of surfactant deteriorates the surface tension of the base fluid and increases the suspension time of particles in the liquid [33]. Asadi et al. [34] investigated the impact of sonication time (10 – 160 min) and surfactant (Cetyltrimethylammonium bromide (CTAB), Sodium dodecyl sulfate (SDS), and Oleic acid) on the stability and thermo-physical properties of Mg(OH)_2_/water nanofluids. The nanofluid prepared using CTAB as surfactant showed better dispersion for a sonication time of 30 min. Xian et al. [35] evaluated the impact of ultrasonication time and surfactant on the stability and thermo-physical properties of (COOH-Gnp)-TiO_2_ nanofluids. The experimentation disclosed that CTAB is a better surfactant than SDBS for the present case. The hybrid nanofluid was found to be stable after ultrasonication of 90 min.

Tiwari et al. [36] explored the stability and viscosity of CeO_2_-MWCNT hybrid nanofluid as a function of the base fluid, sonication time, and surfactant. The Benzalkonium chloride (BAC) surfactant was found to be more effective than Potassium lauryl sulfate (PLS), Ammonium lauryl sulfate (ALS), and Cetrimonium chloride (CTAC) for various used base fluids. The optimum sonication time for water, ethylene glycol, silicon oil, and Therminol VP-1 based nanofluids was 90 min, 120 min, 60 min, and 120 min, respectively. In another study, Tiwari et al. [37] examined the impact of surfactant type, surfactant mixing ratio, and sonication time on the stability of CeO_2_ + MWCNT/water nanofluid. The results showed that the nanofluid prepared using CTAB as surfactant has better stability up to 30 days, beyond which Sodium dodecylbenzene sulfonate (SDBS) surfactant-based nanofluid depicted better stability. The optimum sonication time of nanofluid was 90 min, similar to the study [36].

The Fe_3_O_4_ nanofluid prepared using tetramethylammonium hydroxide (TMAH)) depicted better stability than polyvinylpyrrolidone (PVP) and SDS [38]. The SDS based Fe_3_O_4_ nanofluid displayed more enhancement in thermal conductivity. Mostafizur et al. [39] investigated the impact of various surfactants, including CTAB, SDS, and Gum Arabic (GA), on the stability of alumina-methanol nanofluid. The CTAB as a surfactant provided more stabled alumina-methanol nanofluid than SDS and GA surfactants. From the literature, it can be inferred that the sonication time and surfactant can play an essential role in nanofluid stability, which may vary from one type of nanoparticle to another type.

Nanofluids can be used for solar spectrum splitting for various applications [40]. Huaxu et al. [41] used ZnO nanofluid in spectral splitting for photovoltaic systems. Fernandes and Schaefer [42] used various nanofluids as optical filters for Si, GaAs, and GaInP/GaAs solar cells. The efficiency of solar cells improved significantly when nanofluids were employed as spectrum splitters. Sajid and Bicer [5] investigated the effect of spectrum selective nanofluids on the cooling load of a greenhouse. It was found that a cooling load reduction of 26 % for the summer season and a greenhouse set temperature of 292 K is achievable without any significant compromise on the visible spectrum, which is necessary for the photosynthesis of plants.

If nanofluids are being used as spectrum splitters, identifiying how various factors will affect their optical properties becomes vital. The optical characteristics (mainly; transmittance and absorbance) of nanofluids are directly related to the efficiency of solar-based systems (solar collectors and solar stills etc.). That’s why any variation in these properties will change the efficiency of solar-based systems. The present work aims to investigate the impact of ultrasonication time, nanoparticles concentration, and surfactants on the stability and optical characteristics of CuO/water, Fe_3_O_4_/water, and CNTs/water nanofluids. Many researchers studied the optical properties of various nanofluids. Still, according to the author's best knowledge, no effort was made to evaluate the effect of ultrasonication time, temperature, time duration after preparation, and surfactant on the transmission and absorption characteristics of the selected nanofluids, as mentioned earlier. In this regard, the specific objectives of this study can be defined as:

– To prepare CuO/water, Fe_3_O_4_/water, and CNTs/water nanofluids having concentrations of 0.004 and 0.0004 vol% with and without surfactants.
– To investigate the impact of various surfactants and ultrasonication time on the stability of prepared nanofluids.
– To examine the effect of nanoparticle concentration, ultrasonication time, time duration after preparation, and temperature on the optical properties of nanofluids.
– To investigate the spectral irradiance being transmitted through prepared nanofluids in the visible and near-infrared region.

## 2 Material and Methods

### 2.1 Materials

#### 2.1.1 Nanoparticles

The specifications and manufacturer of nanoparticles used in the present work are summarized in Table 2.

**Table 2:** Specifications of nanoparticles used in the present study.

Nanoparticles	Color	Size (nm)	Density (kg/m^3^)	Manufacturer
CuO	Black	<50	6400	Sigma Aldrich
Fe_3_O_4_	Black	50–100	5200	Alfa Aesar
CNTs	Black	Dia: 10–20	2100	Chengdu organic chemicals Co. ltd.

#### 2.1.2 Surfactant

The use of surfactants to enhance the stability of nanoparticles in the base fluid is a common and economical method. The addition of surfactant reduces the surface tension of the base fluid, which as a result, increases the suspension time of nanoparticles in the base fluid. Three types of surfactants, anionic (SDBS), cationic (CTAB), and polymer (GA), are used during the preparation of nanofluids. All tested surfactants were purchased from Sigma-Aldrich. Nanoparticles and surfactants have the same ratio (1:1) during the preparation of nanofluids.

### 2.2 Methods

#### 2.2.1 Preparation of nanofluid samples

Two-step method was adopted to prepare the nanofluids, which is most widely used by the researchers. Initially, the mass of nanoparticles was determined to prepare the specified concentrations (0.0004 and 0.004 vol%) of nanofluids using Eq (1).

(1) $$\emptyset =\left(\frac{\frac{m_{p}}{\rho_{p}}}{\frac{m_{p}}{\rho_{p}}+\frac{m_{bf}}{\rho_{bf}}}\right)$$

where $m_{p}$ is the mass of nanoparticles, $m_{bf}$ is the mass of base fluid, $\rho_{p}$ is the density of nanoparticles, $\rho_{bf}$ is the density of the base fluid and ∅ is the volume fraction. The reasons behind the selection of these specific concentrations include (i) lower concentrated nanoparticles have a lower tendency to agglomerate as compared to highly concentrated nanofluids, (ii) marginal increase in the viscosity, which means less increase in pumping power, (iii) lower cost due to small quantities of particles still keeping the desired features, (iv) nanofluids with lower concentrations have better spectral transmission and made them a preferable candidate for spectrum splitting applications (requiring UV/visible spectrum). The higher concentrated nanofluids will have poor transmittance, thus, making them a poor candidate for spectrum splitting applications.

After calculating the required mass, a highly precise weight balance (EX225D, OHAUS, USA) (with repeatability of 0.015 mg) was used to measure the mass of nanoparticles. The surfactant was dissolved in DI water using a magnetic stirrer. Then the measured amount of nanoparticles was added to DI water. This mixture was subjected to a probe-sonicator (Qsonica, USA) for nanoparticle segregation and uniform dispersion under various periods (30, 60, and 90 min).

#### 2.2.2 Stability

The stability of nanoparticles in the base fluid can considerably influence the thermophysical and optical properties. Many studies evaluated the stability of nanofluids using the zeta potential method. The nanofluids with zeta potential values between 40 and 60 mV or −40 to −60 mV are considered good stabled fluids [33]. (Zetasizer Nano ZSP (ZEN5600), UK) was used to find the zeta potential values of prepared nanofluids. The instrument has a conductivity accuracy of ±10. Three zeta potential measurements were taken for a sample, and an average value is used in the results.

#### 2.2.3 Transmittance and absorbance

Water is used as base fluid in the present work, which has very high transmittance in the visible spectrum and is opaque to the radiations having a wavelength greater than 1400 nm [40]. The addition of a small quantity of nanoparticles can enhance the ability of water to absorb some or complete portion of the visible spectrum. The optical characteristics of nanofluids with various concentrations and surfactants in UV, visible, and near-infrared regions (280 – 1600 nm) were explored using a UV–VIS-NIR spectrophotometer (UV 3600 plus, SHIMADZU, Japan). The wavelength accuracy of the spectrophotometer is ±0.2nm in the UV and visible region, while ±0.8nm in the near-infrared region. A quartz cuvette with a width of 10 mm was used during measurements, and an empty quartz cuvette was used as a reference.

#### 2.2.4 Transmittance variation with temperature

It is crucial to know the effect of temperature on the optical properties of nanofluids because when nanofluids are used in spectrum splitting applications, their temperature will rise due to continuous exposure to sunlight. The nanofluids samples were heated at a temperature of 55°C in a constant temperature bath (Grant Instruments ltd., SAP12) which has an accuracy of ±0.2°C for 6h. The optical transmittance of nanofluid samples was measured before and after heating.

#### 2.2.5 Transmittance variation over time

The nanoparticles start to aggregate after some time, which leads to an increase in the transmittance of nanofluids. It is necessary to evaluate the effect of time duration on the transmittance of nanofluid. Any considerable variation in the optical properties of nanofluid will make it a poor candidate for spectrum splitting application as it will be unreliable. The literature reveals that nanoparticles agglomerates in the early days after preparation. So, the transmittance of prepared nanofluid was investigated after a week of their preparation.

#### 2.2.6 Spectral irradiance

The spectral irradiance being absorbed by nanofluid is directly related to the photo-thermal conversion performance. The surfactant, ultrasonication time, and nanoparticle type can significantly impact the spectral irradiance being absorbed or transmitted through the nanofluid. The spectrophotometer (Ocean HDX, from Ocean Insight, USA) is used to measure the spectral irradiance (μ W/cm^2^/nm) being transmitted through nanofluid in the mainly visible spectrum (400 – 800 nm). For the near-infrared region (900 – 2400 nm), the spectral irradiance transmitted was measured by spectrophotometer (NIR Quest, from Ocean Insight, USA). The halogen lamp (HL-2000-FHSA, from Ocean Insight, USA) was used as a light source during the experimentation.

## 3 Results and discussions

### 3.1 Effect of sonication and surfactant on stability

**Fig. 1:**
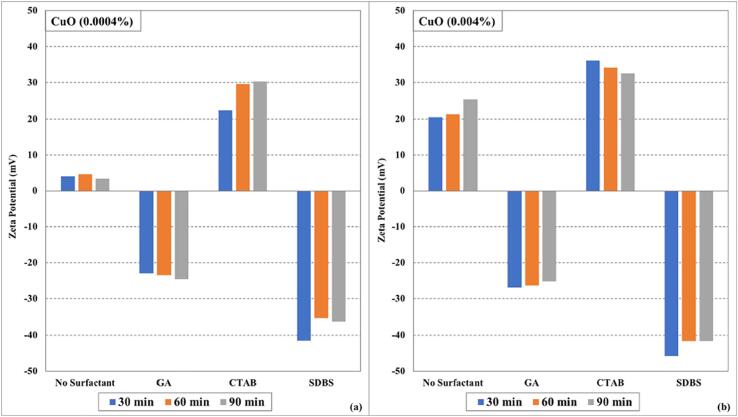
Zeta potential values as function of ultrasonication time and surfactants for (a) CuO (0.0004%) nanofluids (b) CuO (0.004%) nanofluids.

**Fig. 2:**
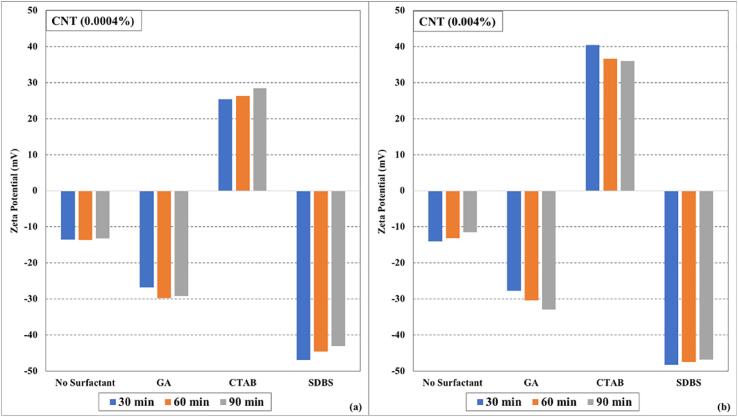
Zeta potential values by varying ultrasonication time and surfactants for (a) CNTs (0.0004%) nanofluids (b) CNTs (0.004%) nanofluids.

**Fig. 3:**
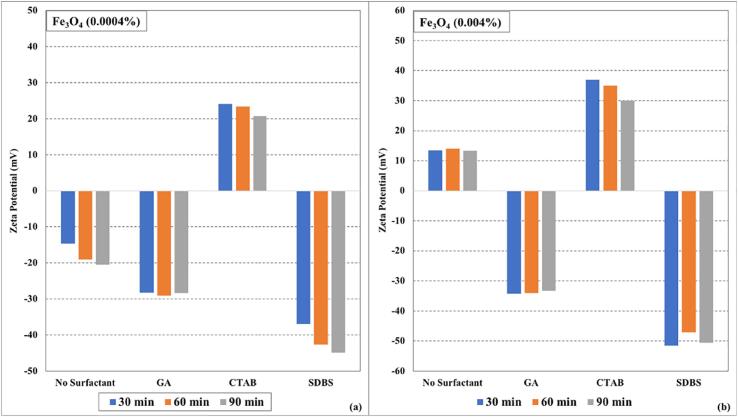
Zeta potential values under influence of ultrasonication time and surfactants for (a)Fe_3_O_4_ (0.0004%) nanofluids (b)Fe_3_O_4_ (0.004%) nanofluids.

It can be observed from the graphs (Fig. 1, Fig. 2, and Fig. 3) that the surfactants seem to have a significant effect on the stability of (CuO, CNTs, and Fe_3_O_4_/water) nanofluids instead of ultrasonication time. The electrostatic repulsion forces start to dominate over van der Waals forces due to surfactant coating on the nanoparticles and keeping them segregated. In some cases, slight deterioration in zeta potential values was observed with an increase in ultrasonication time, which may be due to the heat generated during the sonication process (even in the presence of cooling of nanofluids during the sonication process). The zeta potential values of (CuO, CNTs, and Fe_3_O_4_/water) nanofluids prepared without surfactants were lower than those prepared using surfactants. Among all the tested surfactants, the nanofluid samples displayed more zeta potential values when SDBS was used as a surfactant. The zeta potential values of higher concentrated (0.004 %) nanofluid samples were more than the lower concentrated (0.0004 %) nanofluid samples.

### 3.2 Effect of sonication and surfactant on transmittance

**Fig. 4:**
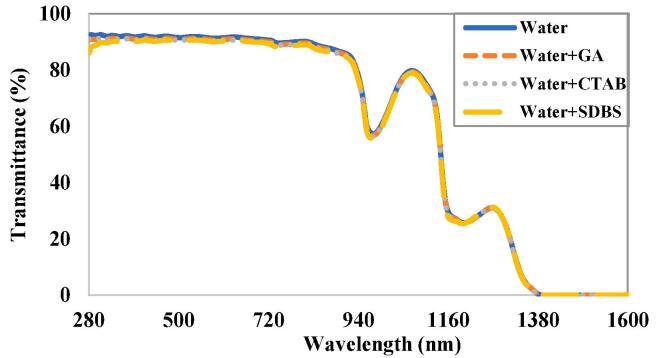
Transmittance of water and surfactants mixed in water.

Fig. 4 illustrates the transmittance of water and various surfactants mixed in water. The amount of surfactant added to water was identical to the maximum quantity of surfactant used during the preparation of nanofluid samples. It is obvious from the graph that the surfactants have a negligible effect on the optical transmission of water. Hence, any change in optical properties of nanofluid compared to water will be purely due to the presence of nanoparticles.

The efficiency of solar systems may be improved by exposing them to a specified window of the solar spectrum. For example, some PV cells have an optimum spectrum window between 700 and 1100 nm. The solar spectrum beside this optimum window will contribute to the reduction in efficiency due to heat generation. So, the optical transmittance of nanofluid can help in the selection of potential nanofluid for a specified application.

**Fig. 5:**
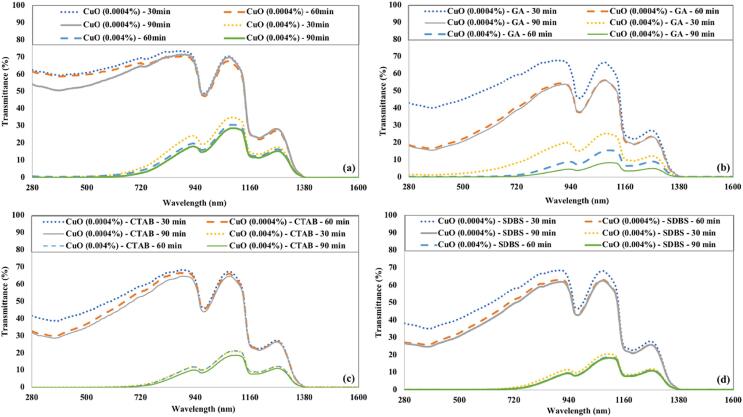
The transmittance of CuO nanofluids by varying ultrasonication time and (a) without surfactant (b) GA (c) CTAB (d) SDBS.

Fig. 5 depicts the transmittance of CuO nanofluids for various ultrasonication times and surfactants. Both concentrations (0.0004 % and 0.004 %) of CuO nanofluids were initially prepared without any surfactant. The transmission of CuO nanofluids prepared without any surfactant was higher than those prepared using a surfactant. It is due to the poor distribution of nanoparticles in the water in the absence of surfactant. The ultrasonication time seems to have less effect on the transmittance of CuO nanofluids having no surfactant. When GA is used as a surfactant, higher sonication times favor nanoparticles' better dispersion (lower transmittance). This phenomenon was more prominent for higher concentrated (0.004 %) CuO nanofluids. For CTAB and SDBS, the optical transmission decreased as the sonication time increased from 30 to 60 min. Further increase in ultrasonication time has no significant effect on the optical transmission of both concentrations of CuO nanofluids. The transmittance of CuO nanofluid having GA as a surfactant was lower than that of CuO nanofluids with other tested surfactants. The CuO nanofluids with a concentration of 0.004 % blocked the visible spectrum, while having some transmittance in the near-infrared region. Initially, a slight decrease and then an increase in the transmittance was noticed for CuO nanofluids with a low volume fraction of nanoparticles (0.0004 %).

**Fig. 6:**
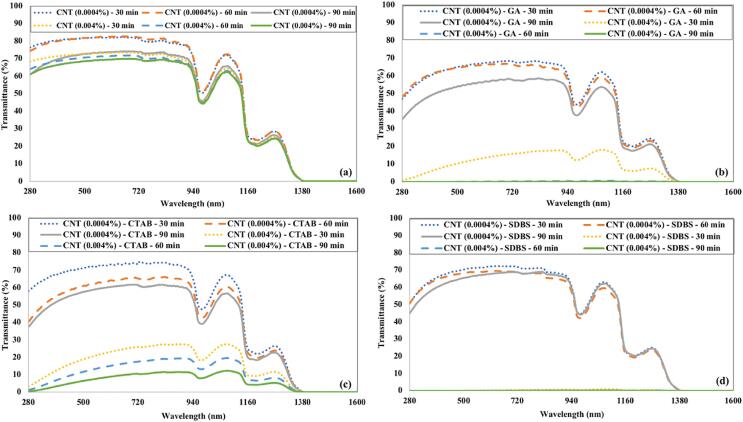
The transmittance of CNTs nanofluids as a function of ultrasonication time and (a) without surfactant (b) GA (c) CTAB (d) SDBS.

Fig. 6 shows the effect of ultrasonication time and surfactant on the transmittance of CNTs nanofluids. The CNTs were not distributed uniformly in the water without any surfactant or even with CTAB as surfactant. Therefore, high transmittance can be observed in both cases. The CNTs dispersion was quick in the presence of SDBS surfactant as the ultrasonication time has no significant effect on transmittance in this case. The distribution of CNTs was also fine in the presence of GA, but it required higher sonication times. The transmittance of CNTs nanofluids has a slight rise and then becomes nearly constant up to 900 nm for low concentration (0.0004 %). In contrast, the high concentration (0.004 %) of CNTs nanofluids (with GA or SDBS surfactant) showed potential to block nearly the whole spectrum (as transmittance is very low).

**Fig. 7:**
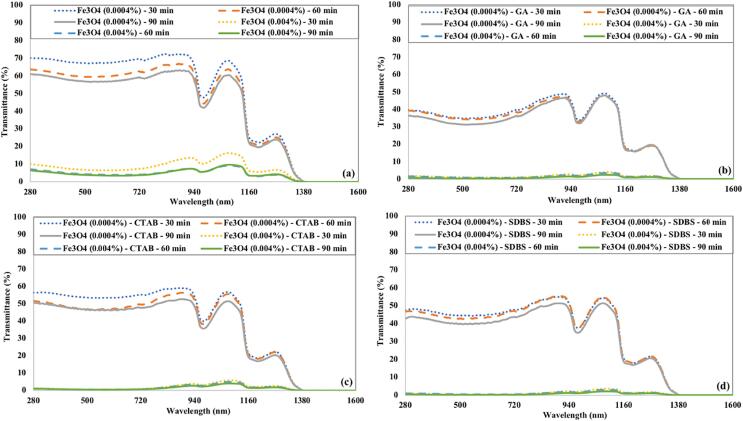
The transmittance of Fe_3_O_4_ nanofluids under the influence of ultrasonication time and (a) without surfactant (b) GA (c) CTAB (d) SDBS.

Fig. 7 illustrates the variation in transmittance of Fe_3_O_4_ nanofluids as a function of ultrasonication time and surfactants. Fe_3_O_4_ nanofluids prepared without any surfactant have the highest transmittance compared to Fe_3_O_4_ nanofluids prepared using surfactants due to poor stability. The Fe_3_O_4_ nanofluid containing CTAB as surfactant showed more transmittance than Fe_3_O_4_ nanofluid having GA or SDBS as surfactant. For similar conditions, Fe_3_O_4_ nanofluids with GA surfactant have the lowest transmittance. The transmittance of low concentrated (0.0004 %) Fe_3_O_4_ nanofluids has a decreasing trend up to 520 nm wavelength, and then it starts to rise again. High concentrated (0.004 %) Fe_3_O_4_ nanofluids are opaque to the visible spectrum with a slight transmittance in the near-infrared region.

### 3.3 Effect of surfactant and sonication on absorbance

The absorbance is a supreme parameter to evaluate the energy capturing potential of some fluid. In the case of some solar systems like solar collectors, solar stills, and solar heating systems, it is desired to absorb the complete solar spectrum to harvest the maximum possible thermal energy. Thus, the details of absorbance of nanofluids for the solar spectrum can lead to the selection of a potential candidate for such systems.

**Fig. 8:**
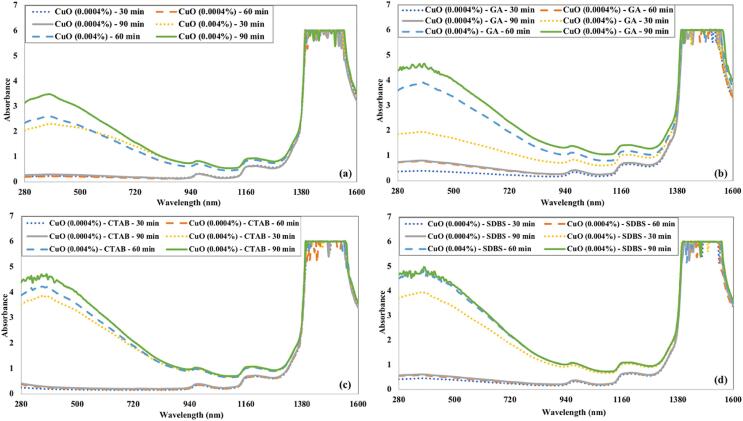
Effect of ultrasonication time and (a) without surfactant (b) GA (c) CTAB (d) SDBS on the absorbance of CuO nanofluids.

Fig. 8 represents the variance in absorbance as a function of ultrasonication time and surfactants for CuO nanofluids. Both concentrations of CuO nanofluids prepared without surfactants have poor absorbance characteristics. The nanofluid will not capture maximum light when the nanoparticles are not uniformly suspended in the base fluid. The absorbance of CuO nanofluids is relatively high in the visible spectrum. However, this absorbance decreased with the increase in wavelength. The absorbance strengthens with the increase in sonication time. CuO nanofluids with SDBS surfactant showed more absorbance. The increase in nanoparticle concentration intensified the absorbance in all cases.

**Fig. 9:**
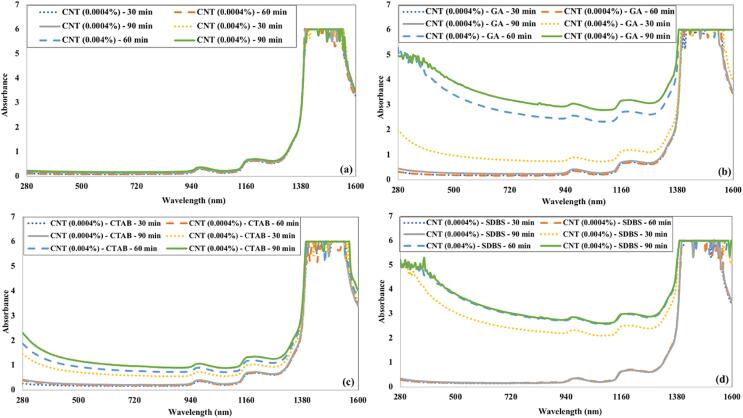
Impact of ultrasonication time and (a) without surfactant (b) GA (c) CTAB (d) SDBS on the absorbance of CNTs nanofluids.

Fig. 9 demonstrates the absorbance of CNTs nanofluids under the influence of ultrasonication time and surfactants. The CNTs remain unsuspended in water in the absence of any surfactant. In this case, the ultrasonication time does not affect the absorbance of CNTs nanofluid. The CNTs dispersed using CTAB as a surfactant also showed poor absorbance characteristics due to poor dispersion. The ultrasonication time slightly impacts the absorbance of CNT nanofluid with CTAB surfactant. The CNTs were homogenously mixed in water in the presence of SDBS or GA as surfactant. The CNT nanofluid showed higher absorbance in the visible spectrum, but it has a significant absorbance in the near-infrared region than CuO nanofluid.

**Fig. 10:**
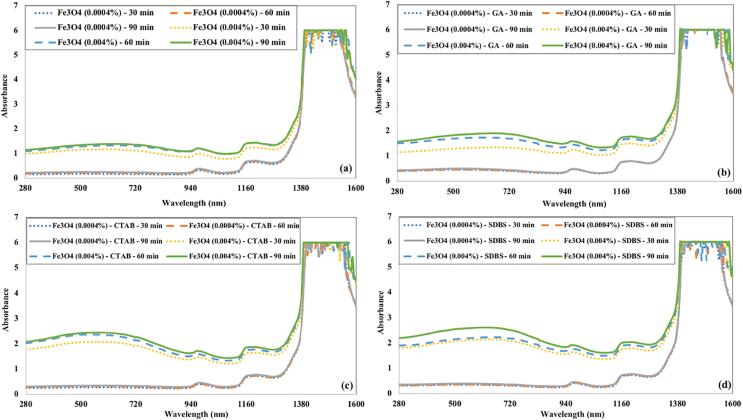
The influence of ultrasonication time and (a) without surfactant (b) GA (c) CTAB (d) SDBS on the absorbance of Fe_3_O_4_ nanofluids.

The absorbance of Fe_3_O_4_ nanofluids as a function of ultrasonication time and surfactants is shown in Fig. 10. The absorbance of Fe_3_O_4_ nanofluids is poor in the visible region compared to CuO and CNT nanofluids in all cases. The absorbance of Fe_3_O_4_ nanofluids prepared without surfactant is slightly lower than that prepared using GA as a surfactant. It is evident from (Fig. 10 (c) and (d)) that Fe_3_O_4_ nanofluids containing CTAB or SDBS surfactants have higher absorbance values. The ultrasonication time does not affect the absorbance of Fe_3_O_4_ nanofluids remarkably like in previous nanofluid cases.

It is clear from the transmittance and absorbance of nanofluids that the samples with 90 min of ultrasonication time have enhanced optical characteristics. Hence, these samples are considered for further investigations.

### 3.4 Effect of temperature on transmittance

**Fig. 11:**
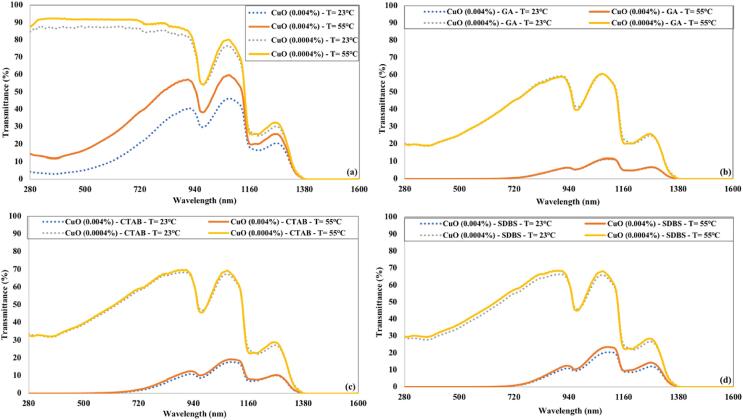
Effect of temperature on the transmittance of CuO nanofluids for 90 min of ultrasonication and (a) without surfactant (b) GA (c) CTAB (d) SDBS.

Fig. 11 depicts the effect of temperature on the transmittance of CuO nanofluids. The increase in temperature significantly impacts the optical transmittance of CuO nanofluid without surfactant. CuO nanofluids prepared using surfactant (CTAB or SDBS) have a minor increase in transmittance. The CuO nanofluid having GA as surfactant showed similar optical transmission for both temperatures.

**Fig. 12:**
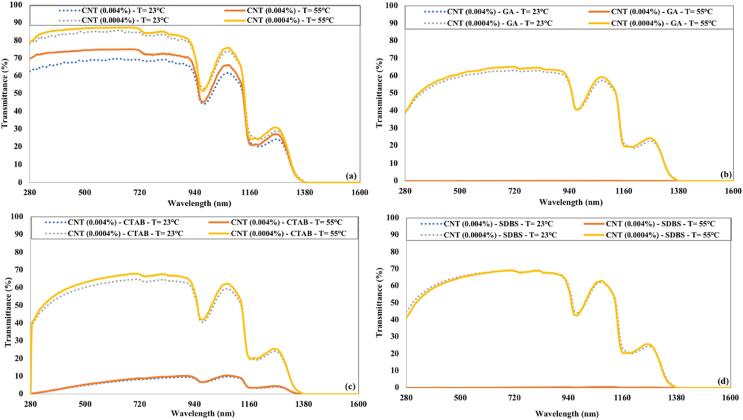
Influence of temperature on the transmittance of CNTs nanofluids for 90 min of ultrasonication and (a) without surfactant (b) GA (c) CTAB (d) SDBS.

The effect of temperature on the optical transmittance of CNTs nanofluids is illustrated in Fig. 12. The transmittance of low concentrated CNTs nanofluid without surfactant was similar to that of water at elevated temperature. The lower concentration of CNTs nanofluids with surfactants displayed a minor increase in transmittance at the higher temperature. The transmittance of higher concentrated (0.004 %) CNTs nanofluids (with GA or SDBS surfactant) remains nearly identical at both temperatures.

**Fig. 13:**
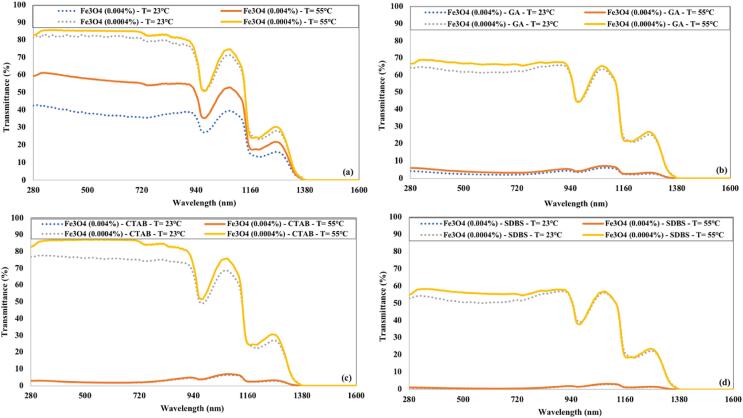
Impact of temperature on the transmittance of Fe_3_O_4_ nanofluids for 90 min of ultrasonication and (a) without surfactant (b) GA (c) CTAB (d) SDBS.

The transmittance of lower concentrated Fe_3_O_4_ nanofluid without surfactant becomes similar to water at a higher temperature, as shown in Fig. 13 (a). The higher concentration of Fe_3_O_4_ nanofluid prepared without surfactant displayed a significant increase in transmittance at 55°C, confirming that nanoparticles sediments at higher temperature in the absence of surfactant. The lower concentrations of Fe_3_O_4_ nanofluids with surfactants have a slight increase in transmittance with the temperature rise. Fe_3_O_4_ nanofluids with a concentration of 0.004 % having surfactant displayed nearly identical transmittance at 23°C and 55°C.

### 3.5 Effect of time on transmittance

**Fig. 14:**
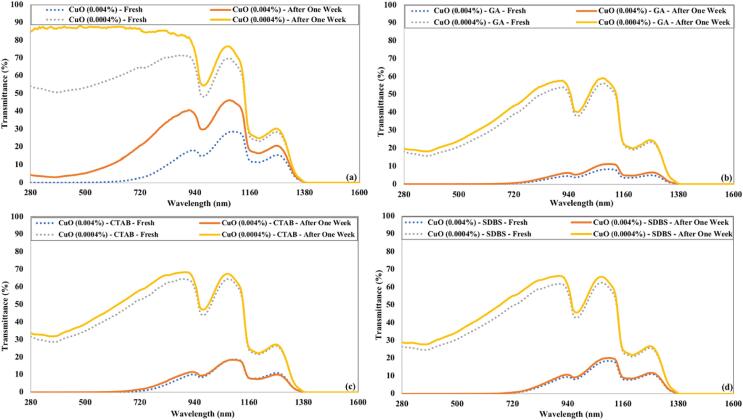
The transmittance of CuO nanofluids just after preparation and after a week for 90 min of ultrasonication and (a) without surfactant (b) GA (c) CTAB (d) SDBS.

The transmittance of freshly prepared CuO nanofluids and after a week of preparation are represented in Fig. 14. The CuO nanofluid prepared without surfactant displayed the highest increase in transmittance after a week of preparation. In the absence of surfactant, the nanoparticles aggregate quickly and settle down. The transmittance of a lower concentration of CuO nanofluid without surfactant becomes identical to that of water. The CuO nanofluids prepared using surfactant depicted a slight increase in transmittance even after a week of preparation. The presence of surfactant keeps nanoparticles segregated and leads to less variation in transmittance over time. The rise in transmittance was more prominent for lower concentrated (0.0004 %) nanofluid than the higher concentrated (0.004 %) nanofluid.

**Fig. 15:**
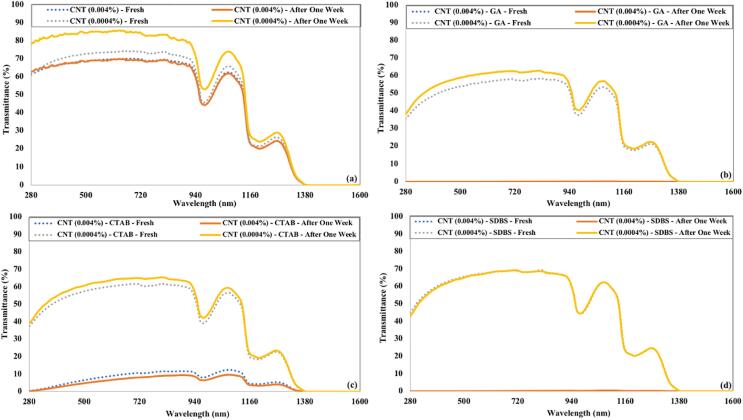
The transmittance of CNTs nanofluids just after preparation and after one week for 90 min of ultrasonication and (a) without surfactant (b) GA (c) CTAB (d) SDBS.

Fig. 15 shows the transmittance of freshly prepared CNTs nanofluids and after a week of preparation. The CNTs were not dispersed in water in the absence of surfactant. That’s why the transmittance of CNTs nanofluids without surfactant was not affected much even after a week of preparation. A slight increase in transmittance of CNTs nanofluid was observed for samples having GA or CTAB as surfactant. The CNTs nanofluids with SDBS surfactant have no increase in the transmittance, which confirms that CNTs are uniformly dispersed even after a week of preparation.

**Fig. 16:**
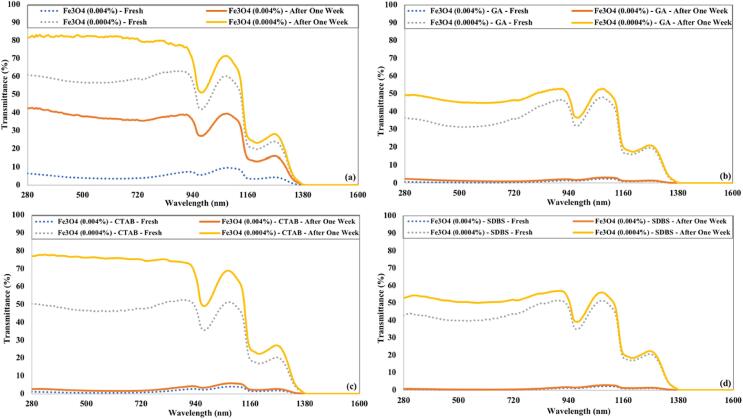
The transmittance of Fe_3_O_4_ nanofluids just after preparation and after a week for 90 min of ultrasonication and (a) without surfactant (b) GA (c) CTAB (d) SDBS.

Fig. 16 illustrates the transmittance of Fe_3_O_4_ nanofluids just after preparation and after a week of preparation. The Fe_3_O_4_ nanofluids transmittance increased significantly for both concentrations prepared without surfactant. For higher concentration (0.004 %) of Fe_3_O_4_ nanofluids, a negligible increase in transmittance was noticed in the presence of surfactant. The lower concentration (0.0004 %) of Fe_3_O_4_ nanofluid having CTAB as surfactant obtained a significant increase in transmittance after a week of preparation.

### 3.6 Spectral irradiance

For spectrum splitting, it becomes essential to know how much irradiance is being transmitted in a specified spectrum region. An effort has been made in this section to investigate the irradiance being transmitted through empty quartz cuvette and cuvette filled with nanofluid in the visible and near-infrared region.

**Fig. 17:**
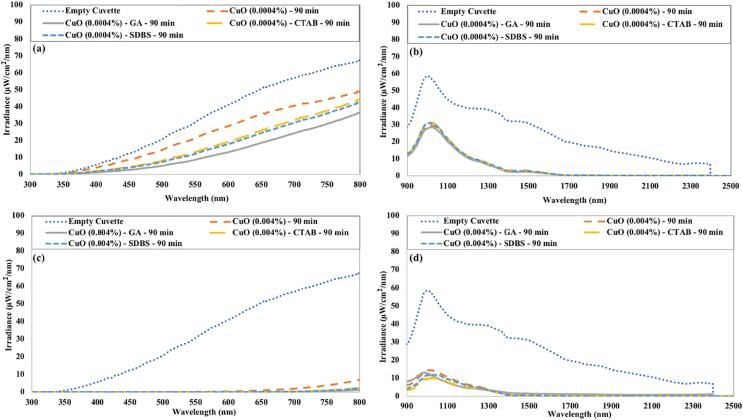
The irradiance transmitted through CuO nanofluids in (a) the visible region 0.0004 vol% (b) the near-infrared region 0.0004 vol% (c) the visible region 0.004 vol% (d) the near-infrared region 0.004 vol%.

The irradiance in the visible and near-infrared region for CuO nanofluids is depicted in Fig. 17. The empty quartz cuvette displayed maximum irradiance transmittance in both (visible and near-infrared) regions. It is evident from Fig. 17 that higher irradiance is being transmitted through low concentrated CuO nanofluids. For a low concentration (0.0004 %) of CuO nanofluid, the effect of surfactant seems more dominating on irradiance being transmitted in the visible region for the same ultrasonication time. While for higher concentrations (0.004 %), this effect is negligible.

**Fig. 18:**
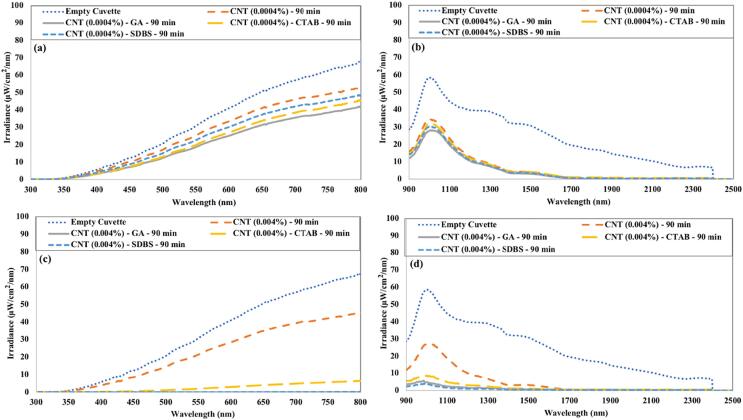
The irradiance transmitted through CNTs nanofluids in (a) the visible region 0.0004 vol% (b) the near-infrared region 0.0004 vol% (c) the visible region 0.004 vol% (d) the near-infrared region 0.004 vol%.

Fig. 18 represents the irradiance transmitted in the visible and near-infrared region for CNTs nanofluids. For low concentrated CNTs nanofluids, SDBS-based nanofluids showed slightly higher irradiance transmittance in the visible spectrum than other surfactant-based nanofluids.

**Fig. 19:**
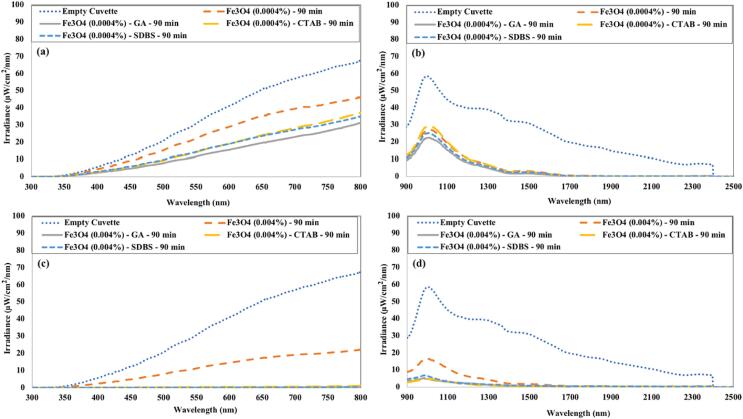
The irradiance transmitted through Fe_3_O_4_ nanofluids in (a) the visible region 0.0004 vol% (b) the near-infrared region 0.0004 vol% (c) the visible region 0.004 vol% (d) the near-infrared region 0.004 vol%.

The irradiance being transmitted through Fe_3_O_4_ nanofluids is displayed in Fig. 19. The Fe_3_O_4_ nanofluid without any surfactant has the highest irradiance transmittance in visible and infrared regions compared to nanofluids with surfactant. For high concentration of Fe_3_O_4_ nanofluid with a surfactant, nearly zero irradiance was being transmitted in the visible spectrum. For Fe_3_O_4_ nanofluid having a concentration of 0.0004 %, nanofluid having CTAB surfactant showed slightly higher irradiance transmittance in the visible spectrum compared to nanofluid having other surfactants.

### 3.7 Repeatability and comparison with literature

**Fig. 20:**
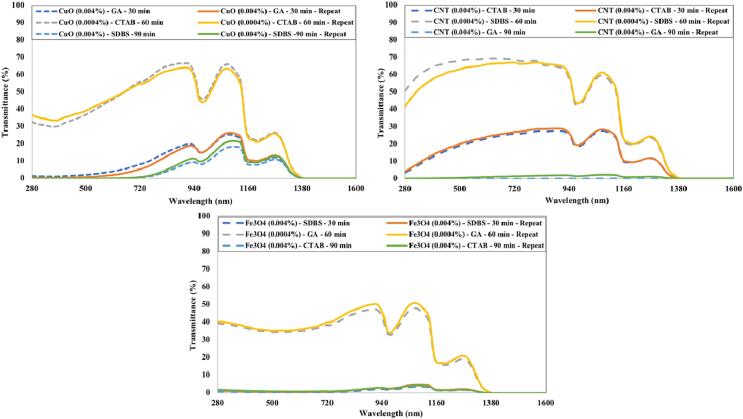
Repeatability of experiments.

The repeatability of experiments is shown in Fig. 20. It is evident from Fig. 20 that the experiments can be replicated with reasonable accuracy (maximum average variation in transmittance observed was between 4.36 % to −1.44 %). It was made sure that the samples should cover all ultrasonication times (30 min, 60 min, and 90 min), surfactants (GA, CTAB, and SDBS), and nanoparticles (CuO, CNTs, and Fe_3_O_4_) during the repetition tests.

**Fig. 21:**
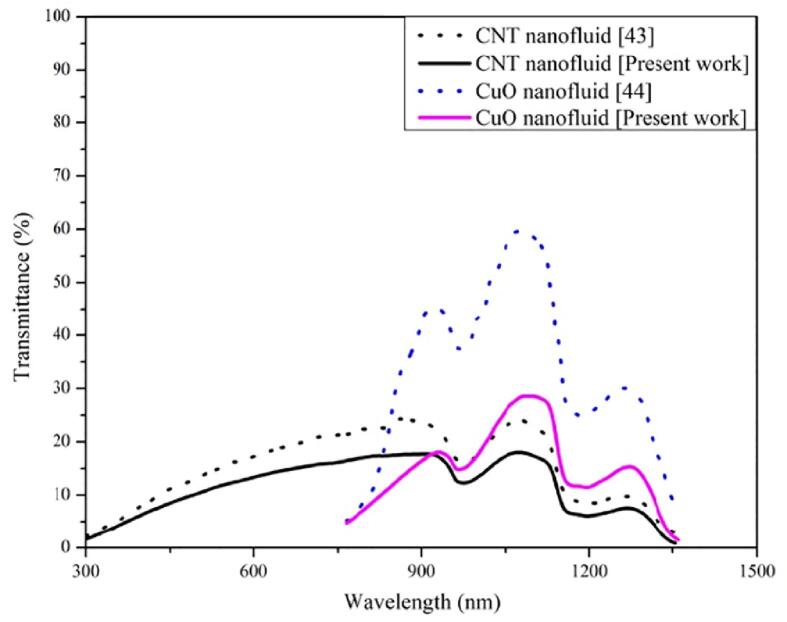
Transmittance of nanofluid for present work and in the literature.

Fig. 21 represents the transmittance of (CuO and CNTs) nanofluids obtained in the present work and from literature [[43], [44]]. It is evident from graph that the transmittance curve trend is same for present and previous works. The transmittance curves obtained in this work is lower compared to previous works due to higher concentration of nanofluids.

### 3.8 Potential applications of prepared nanofluids on the base of optical properties

It is obvious from the optical transmission of CuO nanofluids that they have very low transmittance in the visible region, while high transmittance between 700 and 1100 nm makes them ideal spectral splitting candidates for some types of PV cells and hybrid photovoltaic thermal (PV/T) systems. The CNTs nanofluids (with GA as surfactant) have higher absorbance in the visible region and show better absorbance in the near-infrared region, thus making them a potential candidate for solar collectors, solar thermal systems, and solar stills where maximum solar energy absorption is required. Low concentrated Fe_3_O_4_ nanofluids showed high transmittance in the visible spectrum than CuO and CNTs nanofluids. Their transmittance was significantly lower in the near-infrared region as compared to water. Thus making them potential candidates for greenhouses and buildings to filter the excess solar radiation. As greenhouses require the visible spectrum for photosynthesis of plants and blockage of the near-infrared radiations will help to reduce the cooling load in the hot regions.

## 4 Conclusions

The present experimental study observes the effect of ultrasonication time (30 min, 60 min, and 90 min) and surfactants (SDBS, CTAB, and GA) on the stability and optical characteristics (transmittance and absorbance) of CuO/water, CNTs/water, and Fe_3_O_4_/water nanofluids that can be used in several spectra selective applications. The impacts of nanoparticle concentration, temperature, and time duration after nanofluid preparation on the optical properties of nanofluids were also investigated. The nanofluid samples were prepared with volume fractions of (0.0004 % and 0.004 %) employing two-step method. Based on the obtained results from the experimental work, the following conclusions can be drawn:

• The surfactant has a crucial impact on the stability of nanofluids compared to ultrasonication time for CuO/water, CNTs/water, and Fe_3_O_4_/water nanofluids. The highest stability of nanofluids was observed when SDBS, an anionic type, was used as a surfactant.
• Both ultrasonication time and surfactant showed a significant effect on the optical transmittance and absorbance of nanofluids. With the increase in ultrasonication time, a decrease in transmittance of nanofluids was noticed. This phenomenon was more prominent as ultrasonication time increased from 30 to 60 min for most samples. Among the stabled nanofluids, Fe_3_O_4_ nanofluid displayed higher transmittance in the visible region.
• The absorbance of nanofluids strengthened with an increase in ultrasonication time. The CNTs nanofluids have the highest absorbance among tested nanofluids for 90 min of ultrasonication and SDBS surfactant.
• The temperature rise caused a slight increase in the optical transmittance of CuO/water and CNTs/water (with surfactants) nanofluids having low concentration (0.0004 %). This increase in transmittance was negligible for higher concentration (0.004 %) of CuO/water, CNTs/water, and Fe_3_O_4_/water nanofluids.
• The nanoparticles start to aggregate after preparation, which leads to an increase in the transmittance of nanofluids. Fe_3_O_4_ nanofluids depicted the highest increase in transmittance than other nanofluids.
• The spectral irradiance was found to be a function of nanoparticle concentration and type.

### CRediT authorship contribution statement

**Muhammad Usman Sajid:** Conceptualization, Methodology, Investigation, Writing – original draft. **Yusuf Bicer:** Conceptualization, Methodology, Supervision, Writing – review & editing.

## Declaration of Competing Interest

The authors declare that they have no known competing financial interests or personal relationships that could have appeared to influence the work reported in this paper.
